# Water-sulfur-rich, oxidised adakite magmas are likely porphyry copper progenitors

**DOI:** 10.1038/s41598-023-31736-z

**Published:** 2023-03-28

**Authors:** Timothy S. J. Leong, John A. Mavrogenes, Richard J. Arculus

**Affiliations:** Research School of Earth Sciences, Australian National University, Canberra, ACT 2601 Australia

**Keywords:** Geochemistry, Geology, Petrology

## Abstract

The world’s largest current Cu resource is volcanic arc-hosted, porphyry copper deposits. Whether unusual parental magmas or fortuitous combinations of processes accompanying emplacement of common parental arc magmas (e.g., basalt) is required for ore deposit formation, remains unclear. Spatial and tectonic associations of adakite (andesite with high La/Yb, Sr/Y) with porphyries exist, but genetic links are debated. Delayed saturation with Cu-bearing sulfides consequent to elevated redox state seems essential for late-stage exsolution of Cu-bearing hydrothermal fluids. Partial melting of igneous layers of subducted, hydrothermally altered oceanic crust in the eclogite stability field are invoked to account for andesitic compositions, residual garnet signatures, and the putative oxidised character of adakites. Alternative petrogeneses include partial melting of lower crustal, garnet-bearing sources and extensive intra-crustal amphibole fractionation. Here we demonstrate mineral-hosted, adakite glass (formerly melt) inclusions in lavas erupted subaqueously in the New Hebrides arc are oxidised relative to island arc (and mid-ocean ridge) basalts, are H_2_O-S-Cl-rich, and moderately enriched in Cu. Polynomial fitting of chondrite-normalised, rare earth element abundance patterns shows the precursors of these erupted adakites were unequivocally derived from partial melting of subducted slab, and represent optimal porphyry copper progenitors.

## Introduction

Porphyry copper deposits (PCD) are responsible for ~ 70% of Cu and ~ 25% of Au production worldwide^1^. Although arc magmas are clearly responsible for PCD formation, the genetic details remain elusive. A spatial link between adakite melts and PCD—particularly those rich in gold—has been shown, but the causal link between parental magmas and PCDs remains unclear. Adakites are distinguished from other andesitic magmas by a combination of high Al_2_O_3_ (> 15 wt%), Sr (≥ 400 ppm) and La (≥ 40 ppm), but low MgO (< 3 wt%), Y (≤ 18 ppm) and Yb (≤ 1.9 ppm), leading to high Sr/Y and La/Yb (both > 20)^2^. However, none of these characteristics are fundamentally required of PCD causative melts, making it difficult to understand why such a relationship exists in the first place.

Furthermore, other issues complicate the link between adakites and PCDs:

1. Geochemical analyses of adakites have focused on whole/bulk rocks as opposed to glass inclusions in crystalline phases or glass matrix analyses^3^. Bulk rock compositions differ substantially from original melt compositions due to volatile loss, redox changes, and incorporation of phenocrysts/glomerocrysts/antecrysts/xenocrysts in bulk analyses; 2. High Sr/Y and La/Yb may be attributed variously to fractional crystallization of hornblende^4^ or titanite^5^ instead of garnet. Adakite-like magmas could, therefore, form by partial melting of lower crust (possibly amphibolite ± garnet)^6–8^ or fractional crystallization of these phases from basaltic arc magmas^5,9^. Subsequent to the original assertion that adakites are partial melts of subducted basaltic crust, other tectonic settings have been invoked^10^. This includes: back-arc basins^11^, subduction initiation^12^, subduction termination^13^, oblique subduction^14^, mantle plume-subduction zone interactions^15^, slab tearing^16^, ridge subduction^17^, or shallow subduction^18^. Plausibly, any slab subjected to sufficiently high temperatures may produce adakitic melts.

This study is the first to address the potential PCD-forming fertility of adakite through analysis of glass (formerly melt) inclusions (hereafter abbreviated as MI). Melt inclusions trapped during crystal growth become isolated from the rest of the ambient magma at a moment in time^19^, and may preserve their compositions as closed systems^20^. Consequently, their compositions remain largely unaffected during subsequent crystallization, crystal accumulation and contamination events affecting the host magma. Polynomial fitting of the shapes of chondrite-normalised, rare earth element abundance patterns^21^ of the MI studied here shows unequivocally a critical role for garnet rather than amphibole or titanite in the genesis of the magmas. The pre-eruptive entrapment of MI is also demonstrably favourable for volatile content preservation^22^ and reveals high H_2_O (≤ 6 wt%), S (300–3000 ppm) and Cl (460–3740 ppm) contents, and elevated redox (+ 1.9 to + 3.2 log_10_ oxygen fugacity, relative to the fayalite-magnetite-quartz [FMQ] buffer) relative to basalts. Additionally, Cu contents (674 ppm) exhibit a wide range (Table 1, Supplementary File).

**Table 1:** Results table of melt inclusion compositions.

Sample NLD	0101-71 primary	0101-92	0301-32	0301-41	0301-42	0301-43	0301-44	0301-45	0301-46	0301-51	0301-52 evolved	0301-53	0301-61	0301-62	0301-63	0301-81	0401-61
SiO_2_	58.75	67.50	52.55	53.85	54.86	52.73	53.64	60.65	51.29	55.16	56.40	50.42	57.83	57.36	54.20	54.13	58.91
TiO_2_	0.40	0.62	0.60	0.68	0.84	0.69	0.76	0.39	0.64	0.74	0.67	0.86	0.71	0.60	0.69	0.79	0.39
Al_2_O_3_	19.45	14.88	13.69	15.24	17.34	15.25	15.83	17.95	14.21	14.83	14.56	13.91	19.71	15.44	15.43	19.30	18.39
FeO*	4.27	4.93	8.31	5.79	6.07	7.65	6.94	3.45	8.11	4.68	4.05	5.66	3.55	3.22	4.34	6.78	7.71
MnO	0.13	0.07	0.18	0.09	0.18	0.11	0.16	0.09	0.20	0.05	0.09	0.11	0.13	0.03	0.08	0.16	0.14
MgO	1.89	0.50	2.46	2.18	1.26	2.76	1.77	0.54	2.73	2.12	2.17	3.09	1.35	1.52	2.26	2.20	1.31
CaO	5.71	4.04	14.16	13.82	11.29	12.52	11.78	5.98	14.49	12.50	13.11	14.67	5.91	12.62	13.78	7.17	8.66
Na_2_O	3.40	4.44	1.47	1.73	2.12	1.51	1.40	2.90	1.47	1.69	2.22	1.56	2.50	2.04	1.73	2.48	1.50
K_2_O	0.54	1.32	1.13	1.38	1.84	1.24	1.71	2.70	1.24	1.95	1.49	2.28	2.15	1.81	1.63	2.13	0.39
P_2_O_5_	0.15	0.19	0.45	0.51	0.62	0.56	0.54	0.53	0.52	0.89	0.68	1.12	0.87	0.71	0.60	0.79	0.06
SUM	94.69	98.49	95.00	95.27	96.42	95.01	94.54	95.17	94.89	94.61	95.44	93.67	94.71	95.36	94.74	95.92	97.46
H_2_O (VBD)	5.31	1.51	5.00	4.73	3.58	4.99	5.46	4.83	5.11	5.39	4.56	6.33	5.29	4.64	5.26	4.08	2.54
S	1033	434	2192	2811	611.6	890	658.4	1326	2358	2983.6	2274	3008	1389	2526	2527	1438.4	270
Cl	2005	2545	1028	1290	1226	1187	1809	2245	940	972	3740	1536	1519	960	930	1573	463
Cu	128	114	97	85.9	178	223	556	146.9	272	33.9	10.2	104.3	101	200	115	177	226
Rb	11.4	17.9	33.5	25.1	32.6	27.8	32	25.3	23.1	48.8	48.1	31.3	34.6	40.2	21.1	54	5.3
Sr	473	201	1135	1448	803	1324	1216	1009	1239	2084	2307	1253	1400	1194	1236	1768	111.5
Ba	166	175	318	350	397	244	493	256	329	433	430	357	350	310	309	491	115
Ta	0.49	0.4	0.03	0.02	0	0.05	0	0.03	0	0.22	0.113	0.124	0.26	0.13	0	0	0
Nb	1.34	2.9	1.89	2.27	1.8	2.57	1.9	2.43	2.4	4.1	3.55	3.39	2.9	2.1	2.42	3.2	0.15
Zr	71	163	107.8	118.5	126	117.3	113	108.7	123	221	217.3	110.6	129	108.3	107	152.2	8.7
Hf	2	2.9	1.44	2.9	3.7	2.33	4	3.19	1.81	4.5	5.05	2.98	3	2.5	2.56	3.7	0
Y	12.7	18.3	15.9	17.6	17.3	21.8	21.6	20.5	19.9	29.3	28.3	18	20.7	19.3	17.7	17.9	8.2
La	9.6	10.9	48.8	58.2	69.9	63	52	58.3	54	141.5	128	64.7	69.1	53.2	58	81	0.53
Ce	26.8	34.2	113.4	134.1	153.9	141.8	130	136.3	122	338	302.2	142.7	154	130	126.9	168.4	2.66
Pr	3.49	2.98	13.9	16.4	21.1	17.5	18.1	17.9	13.7	41.5	38.6	17.4	19.2	16.7	16.1	19.6	0.04
Nd	11.4	15.5	57.4	60.7	74	67.3	52	70.6	66	169.7	154.9	74	66	64.6	65.8	75.4	2.5
Sm	4.8	1.2	10.5	11.4	14.8	15.6	13	12.4	12.7	23.6	25.6	11.8	15.3	10.2	11.4	12.2	0
Eu	1.1	1	2.51	3.3	4.3	3.04	4.5	3.58	3.9	6	6.59	3.32	4.2	1.96	2.92	2.09	0.56
Dy	2.4	4.5	6.9	6.5	8.1	8.3	16	7.6	7.7	15.5	16.3	7.2	7.9	5	6.1	6.2	0.24
Gd	0.22	44	0.47	0.65	0.85	0.86	0.88	0.81	0.66	1.67	1.59	0.95	0.75	0.85	0.5	0.54	0.21
Tb	5.1	1.9	4.3	2.9	3.5	5.3	5.2	3.33	4.8	10.4	7.9	4.5	3.6	3.5	3.9	2.7	3.1
Ho	0.94	0.72	0.3	0.65	0.8	0.92		0.73	0.72	1.15	1.03	0.64	0.46	0.46	0.68	0.46	0.72
Er	1.07	1	2.04	3	1.1	1.64	2.1	1.89	1.56	2.46	2.56	1.22	1.43	1.9	1.53	1.49	1.14
Tm	0.21	0	0.21	0.26	0.25	0.17	0.5	0.24	0.26	0.36	0.23	0.139	0.25		0.16	0.18	0.23
Yb	0.69	0.16	1.18	1.8	1.8	1.41	3.4	1.37	2	1.7	1.07	1.79	1.8	1.6	1.7	1.5	0.4
Lu	0.16	0.23	0.2	0.01	0.08	0.29	1.2	0.25	0.2	0.25	0.23	0.12	0.22	0.35	0.12	0.1	0.31
Sr/Y	37.24	10.98	71.38	82.27	46.42	60.73	56.30	49.22	62.26	71.13	81.52	69.61	67.63	61.87	69.83	98.77	13.60
La/Yb	13.91	68.13	41.36	32.33	38.83	44.68	15.29	42.55	27.00	83.24	119.63	36.15	38.39	33.25	34.12	54.00	1.33
ΔFMQ			2.25	3.08	3.16	3.14	3.19	1.91	2.40	2.21	2.99		3.07				

## Adakites in the New Hebrides arc

**Figure 1:**
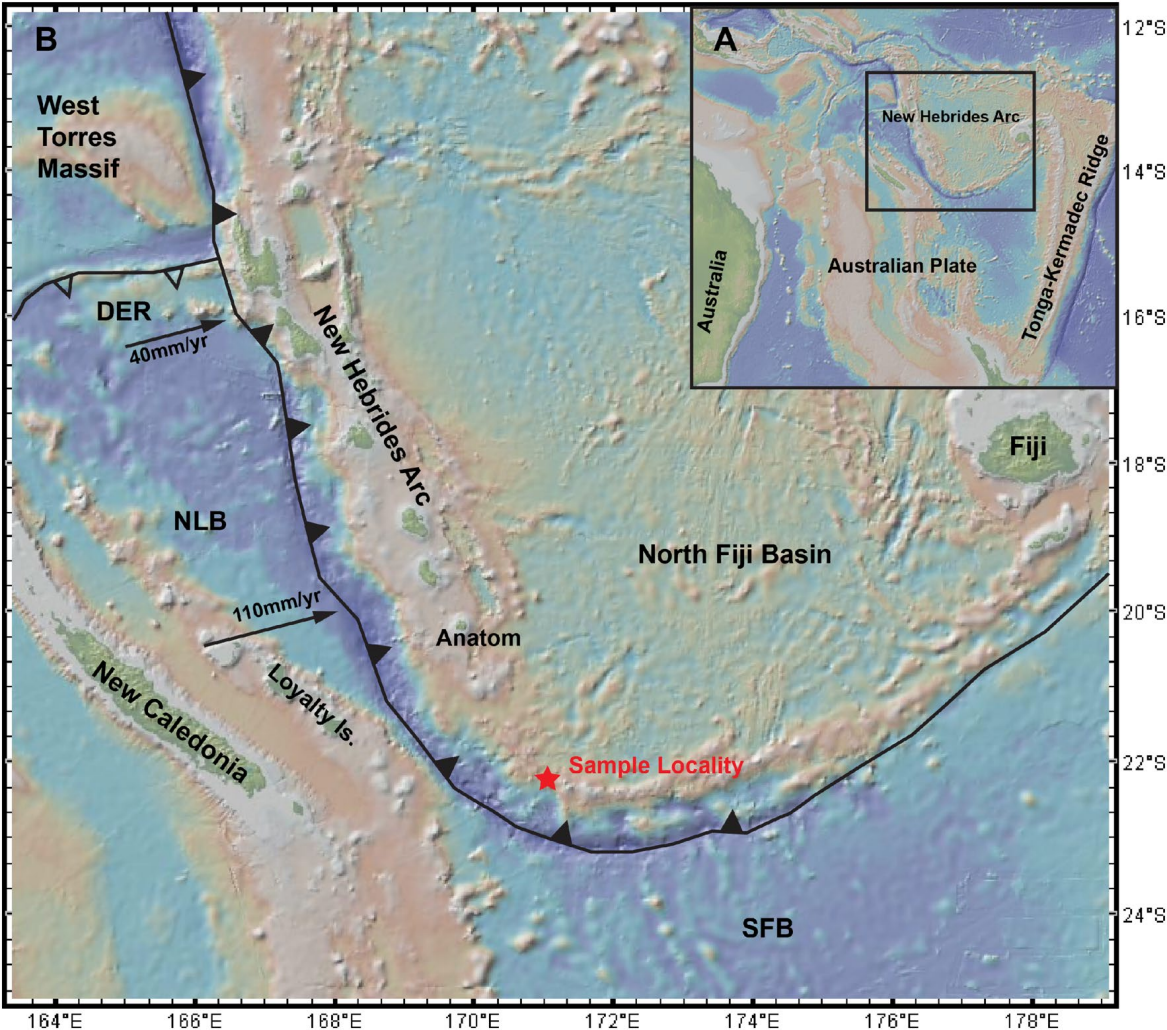
(**A**) Contextual map of the greater region surrounding the New Hebrides Arc. (**B**) Map of the sample site (22.000° S, 170.750° E) in the New Hebrides Arc and the surrounding tectonic systems. Black triangles represent active subduction zones, empty triangles represent extinct subduction zones. *DER* D’Entrecasteaux Ridge, *NLB* North Loyalty Basin, *SFB* South Fiji Basin. Figure made with GeoMapApp v3.6.14 (https://www.geomapapp.org)^30^ (CC BY 4.0). Plate boundaries from Meffre and Crawford, 2001^31^.

The tectonic setting of the New Hebrides island arc is complicated^23–26^. In brief, the Arc on the southwestern margin of the North Fiji Basin is rotating clockwise with accompanying initiation of a new subduction zone as the New Hebrides Trench propagates southeastwards. Along the southernmost portion of the Arc, and along the system of northeast-trending rifts and faults that connect the arc to the Central Spreading Ridge (Fig. 1), a wide variety of magma types, including boninite, tholeiitic basalt, ankaramite, high-Mg andesite and adakite have been erupted^26–29^. Deformation of the subducting slab along its southern termination and toroidal flow of the mantle around the slab edge likely heats the slab^25^ and plausibly triggers localised generation of adakitic magmas.

## Petrology and mineralogy

**Figure 2:**
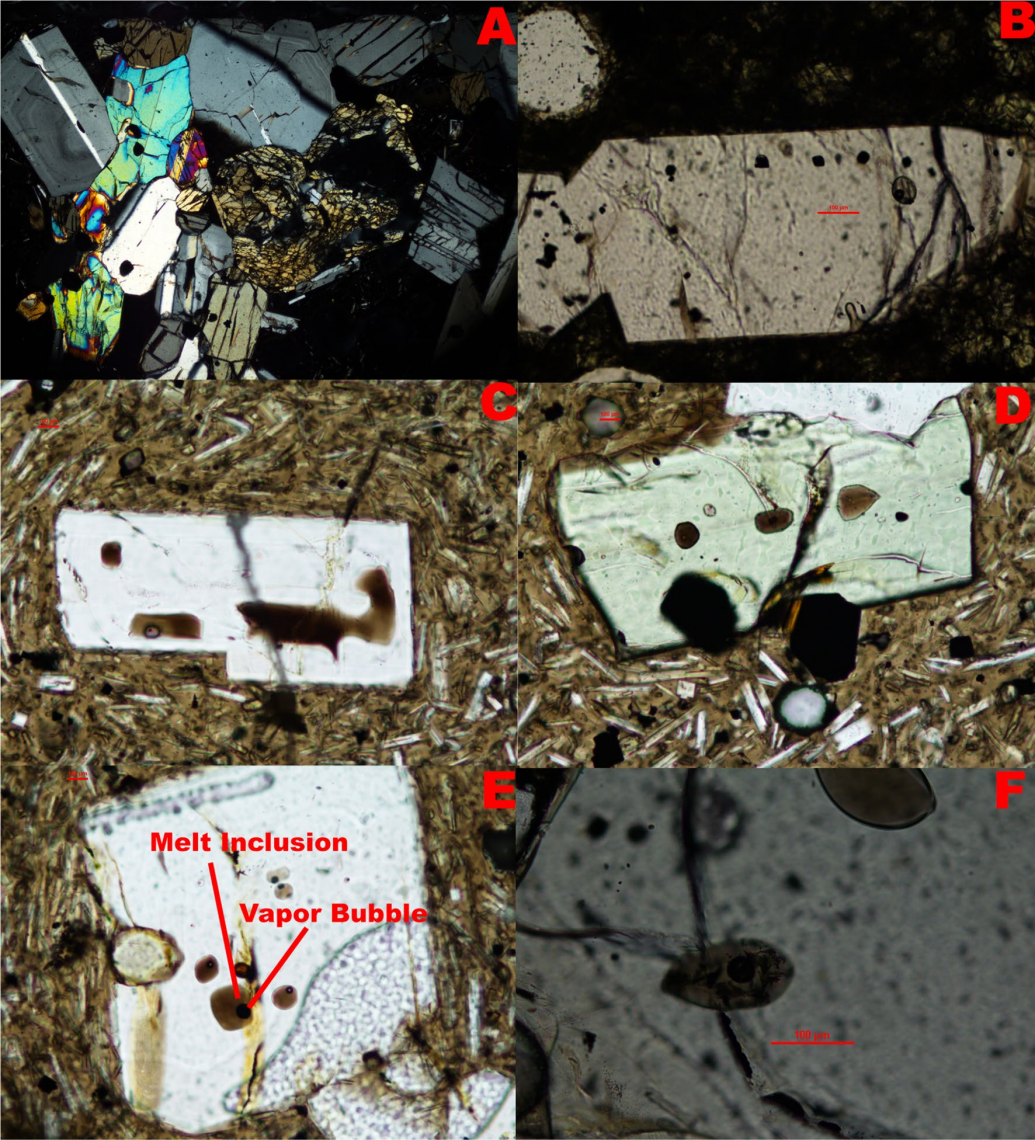
(**A**) Example of a glomerocryst composed of olivine, clinopyroxene and orthopyroxene. (**B**) Small group of melt inclusions. Unlike fluid inclusions, the melt inclusions tended to be a dark brown colour under transmitted light. (**C**) Rectangular melt inclusion and broken melt inclusion that has leaked into the host mineral. (**D**) Irregularly shaped melt inclusions without vapor bubbles. (**E**) Group of melt inclusions with vapor bubbles. (**F)** Large 100 micron melt inclusion.

The MIs studied here are typically 15–35 µm in diameter and hosted in olivine or clinopyroxene phenocrysts within larger olivine-clinopyroxene-orthopyroxene glomerocrysts amongst the plagioclase-dominated matrix (Fig. 2). Most MIs are equant, although olivine also contains melt patches. Plagioclase-hosted MIs are generally of poor quality, being either moth-eaten or in sieve textures, suggesting post-entrapment modification—precluding them from study. Analytical methods and results are presented in the supplementary materials.

## True adakites and how to find them

There are several issues with the current use of the term ‘adakite’. In the absence of crystal-free glass compositions, which are regularly recovered for example from mid-ocean ridge and back-arc settings, the vast majority of ‘adakite’ rocks described in the literature are crystal-rich, a consequence primarily of high dissolved H_2_O contents. Their bulk compositions are likely modified by one or more of: selective accumulation of non-cotectic proportions of crystals in equilibrium with the host melt; incorporation of foreign material from genetically unrelated lithologies encountered en route to the Earth’s surface; and further compromised by volatile loss and redox modification. As an aside, these problems more generally plague phyric volcanic rock classification schemes^32^. A primary reliance on the use of trace element ratios (Sr/Y and La/Yb) for adakite identification may be further undermined by accumulation of crystalline phases such as plagioclase. Although genetic interpretations should not be required for classification purposes, bulk rock Sr/Y and La/Yb are incapable of discriminating between the important petrogenetic processes that require fractionation of garnet during slab melting versus hornblende fractionation at lower pressures, or even low-pressure plagioclase crystallization.

While accepting the general compositional discriminatory parameters for adakite^2^ listed in the opening paragraph, we propose the term only be applied to glass or MI compositions. In the absence of such a constraint, it is difficult to use the term ‘adakite’ in petrogenetically rigorous and useful ways. The prime advantages of this approach—demonstrated by the results of this study—are the quantification of volatile concentrations, enhanced possibility of the preservation of comparatively pristine redox conditions, and to screen samples that have been modified by garnet rather than amphibole fractionation.

The emergence of orthogonal polynomial fitting for chondrite-normalised rare earth element (REE) abundance patterns, and consequent quantification of pattern shapes (described below)^21^, provides a critical tool with which to identify the respective roles of garnet and/or amphibole in the genesis of adakites. Furthermore, the use of MI analyses that are distinguished by high Sr/Y and La/Yb ratios with garnet fractionation vectors from orthogonal polynomial fitting and high temperature estimates from either H_2_O/Ce or two-pyroxene geothermometry, provide a method by which partial melts of subducted slabs can be identified.

## Determination of garnet fractionation through quantification of REE abundance patterns

Plots of chondrite-normalised (e.g., using carbonaceous type I [CI]) REE abundances typically define lines and curves of varying complexity. Qualitative comparisons of the general shapes of these patterns have been commonplace since the 1960s. Rigorous quantification of the shapes by O’Neill^21^ however, has provided a fundamental improvement in our ability to identify the sources, processes and phases involved in the generation and evolution of the spectrum of global magma types. Previously, distinguishing heavy (H)REE fractionation via garnet vs hornblende in magmatic systems has been difficult when based on qualitative comparison of REE abundance patterns, or even more simplistically by La/Yb ratios alone. However, orthogonal polynomial fitting can map REEs to polynomial curves through the equation^21^:

1 $$ {\text{ln}}\left( {\left[ {{\text{REE}}} \right]/\left[ {{\text{REE}}} \right]_{{{\text{CI}}}} } \right) = \, \lambda_{0} + \, \lambda_{1} f_{1}^{orth} + \, \lambda_{2} f_{2}^{orth} + \cdots $$

**Figure 3:**
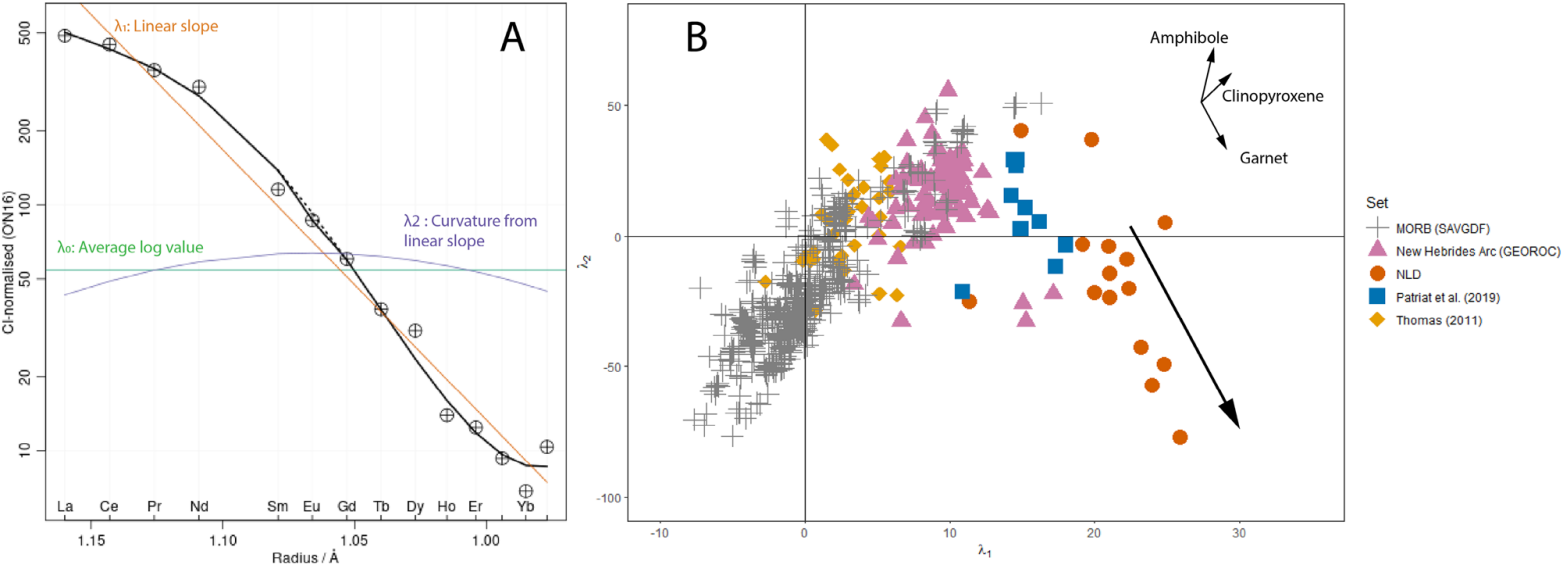
Orthogonal polynomial fitting of REE curves. (**A**) Visualization of how polynomial terms are expressed in relation to an original REE curve. (**B**) *λ*_*1*_ versus *λ*_*2*_*.* The vectors of clinopyroxene, hornblende and garnet fractionation are shown. Garnet fractionation produces a strong and distinct trend towards higher *λ*_*1*_ and low *λ*_*2*_. *λ*_*1*_ and *λ*_*2*_ from: 1. Mid-Ocean Ridge Basalts from the Smithsonian Abyssal Volcanic Glass Data File^33^; 2. Subaerially erupted compositions from Aoba volcano in the New Hebrides arc sourced from the GEOROC database^34,35^; 3. Subaqueously erupted compositions from the Gemini-Volsmar volcanic field^36^ and the Matthew and Hunter subduction ridge^26^ in the New Hebrides arc are provided. MORB compositions provide a baseline against which most fractionation processes can be compared. The REE compositions of the subaerial magmas and Gemini-Volsmar volcanic field magmas fail to display garnet activity, whereas magmas from the Matthew and Hunter subduction ridge show possible garnet activity at source. In comparison, the samples from this study (NLD) not only show clear garnet activity but exhibit strong enough vectors to evidence garnet crystallization.

The utility of this equation is in the independent coefficients, where *λ*_*0*_ is the overall average abundance of the REE of a given sample, *λ*_*1*_ describes the linear slope and *λ*_*2*_ the curvature of the quadratic curve. The numerical values of these coefficients can be used to identify subtle changes in curvature from respective phase fractionations. Most importantly in the current context, garnet fractionation generates a distinct trend in *λ*_*1*_ versus *λ*_*2*_ space, trending to high *λ*_*1*_ and low *λ*_*2*_ values whereas hornblende leads to both high *λ*_*1*_ and *λ*_*2*_ (Fig. 3). The adakite MIs from the southern submarine New Hebrides arc (NLD) define a marked and extensive trend along an unequivocal garnet fractionation vector in *λ*_*1*_ versus *λ*_*2*_ space (Fig. 3). Fractionation of hornblende in contrast would impart a vector with increasing rather than decreasing *λ*_*2*_. The observed effect of the fractionation of garnet from these MIs can be seen relative to the *λ*_*1*_/*λ*_*2*_ compositions of regional samples (e.g., GEOROC sourced whole rock and nearby volcanic rock compositions from the New Hebrides arc; Patriat, Thomas), with MIs tracking along the garnet fractionation vector.

High Sr/Y and La/Yb (averaging > 65 and > 59 respectively) coupled with clear evidence of the role of garnet in HREE fractionation suggest that the MIs are adakites. Both H_2_O/Ce ratios^37^ and two-pyroxene geothermometry^38^, independently yield temperatures of 900–1000 °C ± 20 °C and pressures of 9.2 ± 1.4 kbar, well above the minimum ~ 840 °C required for slab melting^39^. Trace element compositions known to be selectively enriched/depleted in melts in equilibrium with residual rutile eclogite lithologies are consistent with a partial slab melt hypothesis^12^. Further examination of trace element compositions with regards to *λ*_*1*_ show trends of increasing concentrations of incompatible elements. However, no trends were observed relative to *λ*_*2.*_

## Adakite melt inclusions show indicators of fertility

There are three characteristics that are significant in terms of the potential (also termed fertility) of a given magma to generate a PCD: high oxygen fugacity^40,41^, high sulfur contents^42,43^, and high water contents^44,45^. Elevated copper and chlorine contents are beneficial but not necessarily required for PCD formation, with the characteristics outlined above considered more important.

The adakite MIs studied herein have all three characteristics. Firstly, they are more oxidised (+ 1.9 to + 3.2 ΔFMQ) than typical ocean floor basalts (~ + 0.1 ΔFMQ^46^) or many arc rocks. The high *f*O_2_ of adakite melts has previously been attributed to their origins by partial melting of layers 1 and 2 of subducting oceanic crust, involving oxidised terrigenous sediments and altered basalt^40,42^. It is thought that slab melts transport 10^4^ times more Fe^3+^ into the mantle wedge than slab-derived fluids^40^.

Secondly, the sulfur contents of 300–4000 ppm (Table 1, Supplementary) exhibit a wide range, with higher maximum S than typical arc magmas (900–2500 ppm)^47^. High S in arc magmas compared with ocean floor basalts has been attributed to the high oxidation states of the former^48,49^, whereby the more soluble S^6+^ predominates^50,51^ compared with dissolved S^2-^ being the majority in the latter We interpret the wide sulfur range as the result of degassing. Sulfur-poor samples are likely to have experienced sulfur degassing, whereas sulfur-rich samples did not. Whilst the sulfur ranges are not significantly enriched relative to typical arc magmas, they are sufficiently high for PCD generation.

Thirdly, the water contents found here (≤ 6 wt% H_2_O) are within the range deemed necessary for PCD formation^45^. Water in arc magmas derives from the breakdown of hydrous minerals in the down-going slab^52^. Most importantly, the liberation of fluids under the P–T conditions found here can promote slab melting, rather than the release of supercritical fluids commonly associated with typical arc-associated basaltic magmas^53^. Consequently, adakite magmas retain much of the water released from the slab, which is beneficial for PCD generation later on. In fact, most primary arc magmas show no evidence of garnet modulation in their generation^54^.

However, ideas of single stage exsolution of large quantities of fertile fluids from adakite melts during melt evolution are complicated by the behaviour of S, Cl and Cu in the MIs studied (see supplementary). Firstly, S declines sharply at ~ 60 wt% SiO_2_—however, we do not interpret sulfide saturation to be the main vehicle for this decrease as S correlates with H_2_O, being highest at 5 wt% H_2_O but decreasing with lower H_2_O. This is indicative of ascent-driven degassing of a S-rich vapor phase^54^. Secondly, Cl increases steadily with SiO_2_, which could potentially be explained by saturation in CO_2_ well before H_2_O^55^. This leads to high S/Cl ratios in exsolved fluids but low S/Cl ratios in remaining melt^56^, aligning with PCD models that invoke a dense hypersaline brine, and a low-density S-rich vapor^57^. Sulfur exsolves as H_2_S or SO_2_ dissolved in the CO_2_-rich vapor^58,59^. However, the brine carries the vast majority of Cu within due to the stabilizing effect of Cl-ligands on Cu^60,61^. Exsolution of volatiles from adakite parental melts could therefore take the form of cogenetic but temporally distinct pulses of S-rich vapours and Cu-rich brines^62^.

## Implications

Slab melts may play a critical role in the generation of oxidised PCD-forming magmas. While fluids released via dehydration can oxidize the mantle wedge, the degree of oxidation is insufficient for the generation of sulfide under-saturated melts when metasomatized mantle is remelted. Instead, slab melts are likely the main agent of oxidation, carrying up to 10,000 times more Fe^3+^ than slab fluids^40^. Although melts similar to adakite slab melts with high Sr/Y and La/Yb can be generated through melting of the lower crust, such melts would not have the high oxidation or water and sulfur contents needed for PCD formation^63^.

The high oxidation state of slab melts plays a key role in melt fertility with S^6+^ being far more soluble than S^2−^. It is therefore easier for an adakite melt to dissolve sulfur from sulfide residues during initial melting or from immiscible Fe-S–O liquids. Furthermore, oxidised melts retain high sulfur concentrations throughout magmatic evolution by suppressing sulfide saturation^50^. Early sulfide saturation can potentially inhibit melt fertility by rapid chalcophile element sequestration into sulfides^64^. However, with sulfide saturation delayed in oxidised melts, sulfur and chalcophile element concentrations build up during melt evolution. In PCD models where sulfide saturation is not considered detrimental to melt fertility^65^, the introduction of oxidized melts could play a role in the remobilization of metals trapped in previously precipitated sulfides^66^—thereby providing the vehicle for the eventual transport of both sulfur and chalcophile elements upwards.

The water content of adakite melts is an additional contributor to fertility. Water is critical for PCD formation as ore metals partition strongly into fluid phases, stabilised as chloride, bisulfide, or hydroxyl complexes^67^. These complexes form due to the partitioning of Cl^−^ and sulfur (as H_2_S and SO_2_) into fluids. As a result, volatile exsolution triggers the transfer of sulfur, chlorine, and copper into the fluids that drive PCD formation. The water content of adakites is essential for producing the immense quantities of fluids required for PCD formation.

In conclusion, this study shows that orthogonal polynomial modelling of REE patterns can quantitative and unequivocally identify the effects of garnet fractionation. Geothermobarometry shows evidence of high temperatures and pressures sufficient to cross the eclogite solidus in the down going slab. This combination suggests slab melting, an uncommon characteristic of arc magmas^53^. Additionally, this study demonstrates that adakite melts represent plausible progenitors for PCD formation due to their elevated oxidation state, sulfur, water, and chlorine contents all derived from their origin as partial melts of the subducted eclogitic lithosphere. However, further attention should be given to their relationship to PCD formation as it is clear from the lack of PCDs in the New Hebrides that just adakite melt is insufficient to produce PCD—other factors like crustal thickness, tectonic setting and magma volume are required. Ultimately, these findings indicate that the link between adakites and PCDs stems from their slab melt origin and explains why ‘adakites’ found in non-arc settings are rarely associated with PCDs.

## Supplementary Information

Supplementary Information 1.

Supplementary Information 2.

Supplementary Information 3.

## Data Availability

All data generated or analysed during this study are included in this published article and its supplementary information files.
