# Supplementary material for: Water-sulfur-rich, oxidised adakite magmas are likely porphyry copper progenitors
Source: Sci Rep. 2023 Mar 28;13:5078. doi: 10.1038/s41598-023-31736-z (PMC10050068; doi:10.1038/s41598-023-31736-z)
Supplement: Supplementary file 1 — Supplementary Information 1. [file 41598_2023_31736_MOESM1_ESM.docx]

**METHODS**

Samples were obtained from dredges of the Eva, Evita, and La Perouse volcanic seamounts at the southern termination of the New Hebrides Arc during the Australian Marine National Facility voyage SS07/2008^1^. Sample rocks were cut into small pieces and subsequently mounted in resin in one-inch molds. These samples were then polished by hand with diamond-grit sandpaper from grades of 82μm down to 10μm, before being polished on diamond laps down to a grit of 1/4μm.

Quantitative analysis of major and minor elements was carried out with the JEOL 8530F Electron Probe Microanalyzer at a resolution of 20nm at 10kV at 10nA with 5-10μm spot sizes. The high precision (~0.1wt%) of the electron microprobe was also harnessed to provide quantitative element compositions to aid with LA-ICP-MS data calibration. The elements and compounds measured were SiO_2_, Na_2_O, MgO, Al_2_O_3_, K_2_O, CaO, FeO, MnO, TiO_2_, NiO, CuO and P_2_O_5_ as well as S and Cl. All elements were analyzed by their K_a_ x-ray lines.

Trace element analysis was carried out with the aid of Laser Ablation Inductively Coupled Plasma Mass Spectrometry (LA-ICP-MS). The system used was the Lambda Physik LPF Pro 202 192nm laser system equipped with an Agilent 7700 ICP Mass Spectrometer and was located at the ANU Research School of Earth Sciences Jaeger 1 Building. Sample analysis entailed the ablation of melt inclusions using 20μm spot sizes and laser pulses of 5hz frequency. An additional 20 seconds of pre-ablation and of post ablation time were given for each analysis to record background levels. The analyses were calibrated against NIST610 and NIST612 synthetic glass standards, which were measured before and after the analysis of inclusions from each sample mount. Final data reduction processing of data was carried out with the Iolite program hosted on the IGOR6.34A software. The isotopes analyzed were Al^27^ (as an internal standard), S^34^, Cl^35^, Mn^55^, Cu^63^, Rb^85^, Sr^88^, Y^89^, Zr^90^, Nb^93^, Ba^137^, La^139^, Ce^140^, Pr1^41^, Nd^146^, Sm^147^, Eu^153^, Gd^157^, Tb^159^, Dy^163^, Ho^165^, Er^166^, Tm^169^, Yb^172^, Lu^175^. Hf^178^ and Ta^181^. Originally, Ca^44^ was used as the internal standard. However, due to the potential interference of Si^28^ and O^16^, the samples were re-analyzed with Al^27^ as the internal standard. This was possible as Na^23^ and Al^27^ were measured in the original ablations as potential alternative internal standards.

The ablation of sites on samples was complicated by the small size of melt inclusions. As such, some of the analyses were compromised by ablation of the host mineral rather than the melt inclusion. To filter out the compromised results, trace element abundances were normalized against CI chondrite concentrations. Sites that reflected the trace element geochemistry of their host minerals were discarded. Below are examples of how compromised points were discarded, first by laser traverse and then by REE pattern.


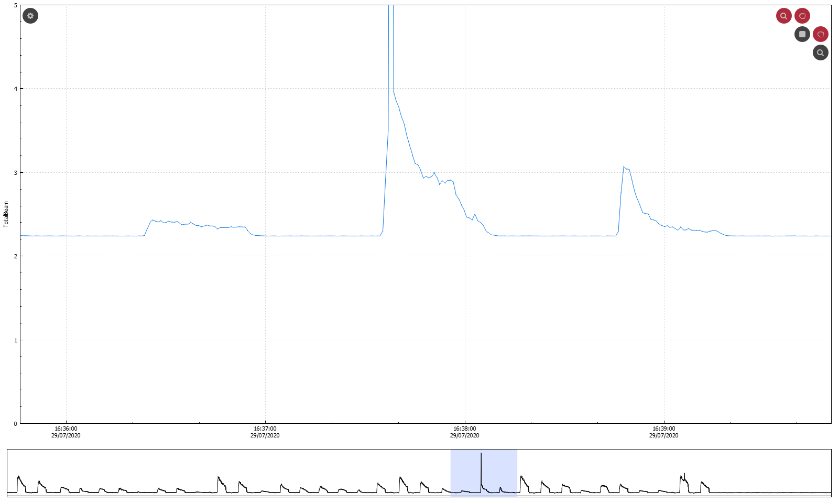
The sharp spike in this sample suggests that foreign phase – potentially a sulfide - was ablated. This sample was then discarded from analysis.


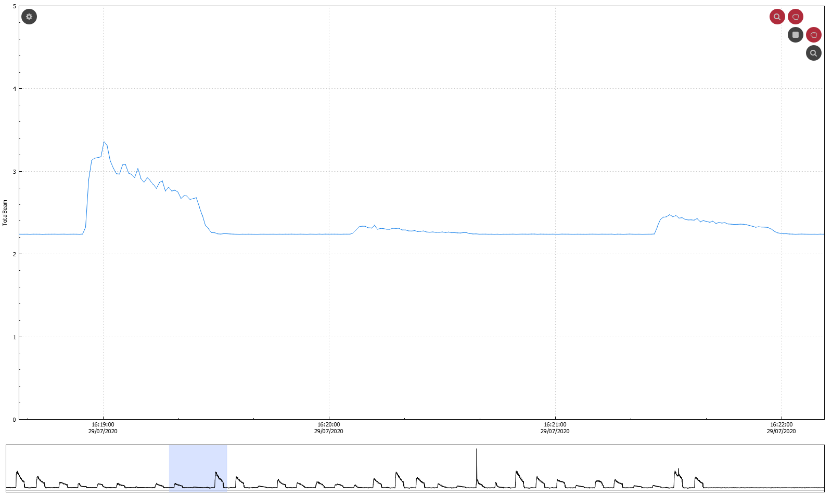
Of these three analyses, the 2^nd^ and third showed low counts. Samples that had such low counts were reflective of points that just hit host olivine


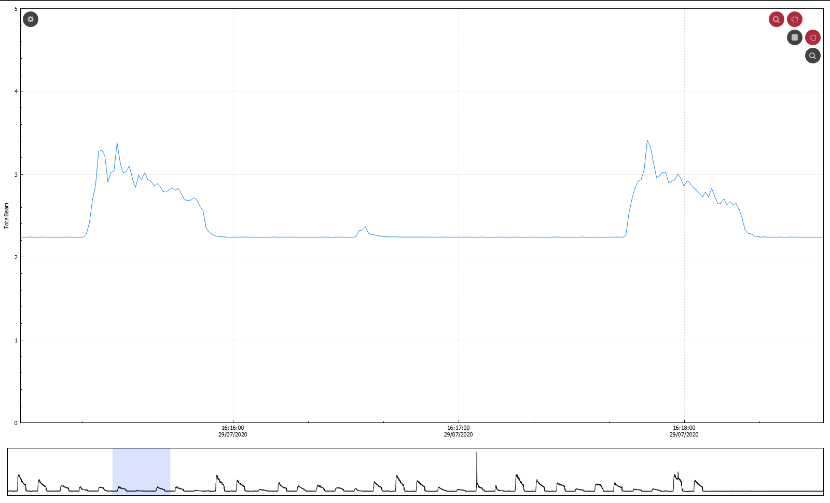

The middle sample barely showed up, and was thus discarded.

For trace element filtering, the BLambdaR program (available at <https://lambdar.rses.anu.edu.au/blambdar/>) was used for initial quality control, with samples that had low r^2^ values removed. Next, the MIs and their hosts tended to have completely different REE patterns. For example, the REE pattern of a host olivine is shown below

_
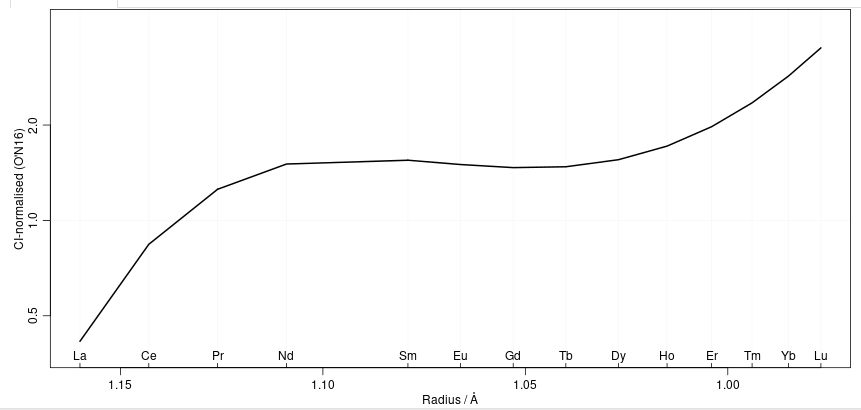
_

In contrast, most MIs had completely different REE patterns
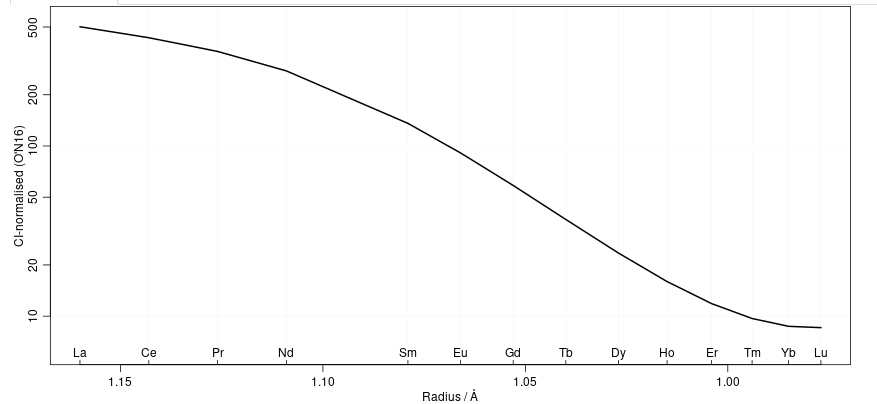
.

Samples that were compromised tended to have 1. Low r^2^ values and 2. strange REE patterns like in the sample below, which had a r^2^ of 0.16.

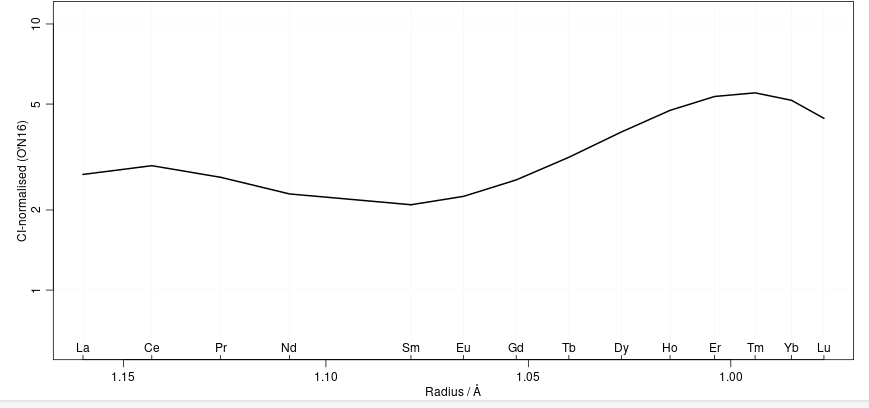


Post entrapment crystallization modelling was carried out using the Olivine MI modelling in the Petrolog3 software^2^ on all the olivine-hosted melt inclusions from the main table, with only columns B,C,Q and R not being modelled as they were hosted in clinopyroxenes. The results of the corrections are available in the supplementary table *“PEC_Output.csv”*, with the host mineral compositions available in sheet 2 of the supplementary file *“supplementary melt inclusion data.xlsx”*. Within *“PEC_Output.csv”,* there are three rows for each melt inclusion. The first row is the original composition (with adjusted Fe2O3 and FeO from the original FeO*). The second row is the composition when the melt-olivine reaches Fe-Mg equilibrium. Finally, the third row is when the melt and olivine reach Fe-Mg equilibrium and FeO* content within the melt inclusion reaches the user-specified value. The degree of host fractionation was found to be between 4-12%. Since this degree of fractionation would not have been high enough to significantly alter trace element compositions beyond the error of the LA-ICP-MS analyses, we have elected to retain trace element compositions.

Concentrations of water were estimated by subtracting total weight values from EMPA analysis from 100%, with the deficit being interpreted as volatile loss from ignition. The estimated water values were supported by Raman spectral analysis. This was done with a Renishaw InVia Reflex Microscope fitted with a 532nm beam housed at the ANU Research School of Physics and Engineering and processed with the Wire 4.1 Renishaw software. To determine water concentrations from the data, an external calibration was first carried out on samples with known water concentrations. The following equation was used for calibration ^3^:

$$C_{H_{2}O}\left( wt\% \right)/[100-C_{H_{2}O}]=A.R_{w/s}$$

*Where C_H2O_ is the water content in wt%, A is a determinable constant, and R_w/s_ refers to the ratio of the areas of the water and silicate Raman peaks.*

Measurements were taken for the silicate band at 100-1400cm^-1^, and for the vibrations of OH and H_2_O groups constituting the water band at 3000-3800cm^-13^. These measurements were then processed to remove the background. Curves were then fitted to determine the areas of the water peaks and of the silicate peaks.

By plotting the $C_{H_{2}O}\left( wt\% \right)/[100-C_{H_{2}O}]$ against *R_w/s_* and using the given standard water concentrations a graph was plotted to determine A (Figure S1).

Figure S1 Raman water calibration graph

Once the calibration curve was generated, the A value of 0.0706 was used for subsequent calculations. By rearranging the original equation, water concentrations were calculated using the following equation:

$$C_{H_{2}O}\left( wt\% \right)=100.A.R_{w/s}/[1+A.R_{w/s}]$$

Figure S2 Raman H_2_O wt% vs Probe H_2_O wt%

As the Raman water estimates were roughly in line with probe totals, the calculated water weights were then cross-referenced with electron microprobe total deficits to estimate the water content of the samples. We did not re-homogenize the samples due to the complications that can arise from (1) dissolution of the host mineral and (2) volatile loss during the re-heating. For these reasons, we suggest that our H_2_O values represent the minimum water budget, with the acknowledgement that water may be sequestered in the bubble. As such, our numbers still show that adakites MIs have enough water to be viable for PCD formation, and could have even more water than we report.

The use of Fe K-edge absorption spectra via XANES is an established method for quantifying the oxidation state of Fe in glasses in phenocrysts^4^. To carry out the XANES analysis, double-polished mounts were sent to the 13 ID-E Beamline facility at the Advanced Photon Source in Argonne, IL., USA. Target sites on the samples were first mapped to locate melt inclusion sites. These maps were then used to select 2μm size sample spots in melt inclusions for analysis. To mitigate the potential effect of beam damage from such a small spot size, the beam flux was reduced by filtering with 100μm thick piece of aluminium foil.

For all samples, the excitation energy was selected using a Si (311) double crystal monochromator (DCM) - resulting in a spectral resolution of 2.5eV. Spectra were recorded for each sample from 7030eV, with a step size of 2.5eV up to 7100eV, 0.1eV from 7100–7130eV, and 2.5eV from 7130eV to 7350eV. A count time of 0.5s was used for each point resulting in a total scan time of three minutes.

The pre-edge spectra was constrained between ~7100-7120 eV and fitted to two pseudo-Voigt peaks^5^. The pre-edge spectra were then filtered to account for crystal contamination that could have arisen owing to the varying depths of the melt inclusions by discarding spectra with irregular and/or overly sharp peaks. Afterwards, the centroid (i.e. intensity weighted average of the pseudo-Voigt peaks) could then be obtained^6^ and used to determine the oxidation state by cross-referencing the centroid eV value against a linear calibration curve.

Figure S3 Calibration curve for Fe3+/ΣFe against pre-edge centroid eV ^4^

**SUPPLEMENTARY RESULTS**

MgO is highest in the low-SiO_2_ samples but decreases with increasing SiO_2_. FeO shows a decreasing relationship with SiO_2_, dropping sharply between 50-60 wt% SiO_2_. CaO exhibits a similar behavior, with sharp decreases in CaO with SiO_2_ below 62 wt% SiO_2_ but a slower rate of decrease after that point. The decreasing concentrations of MgO, FeO and CaO are likely explained by the crystallization of olivine, clinopyroxene and orthopyroxene – phases that are all common in the samples used in this study.

Al_2_O_3_ shows no significant trends and fluctuated between ~10-20 wt%. The absence of a decreasing Al_2_O_3_ trend could be explained by the suppression of plagioclase crystallization common in adakites. Na_2_O increased overall with increasing SiO_2_ but with high variance. K_2_O and P_2_O_5_ exhibits two main trends, with one grouping exhibiting a strong increase between 50-60 wt% SiO_2_ and a slow increase that lasted until 70wt% SiO_2_.


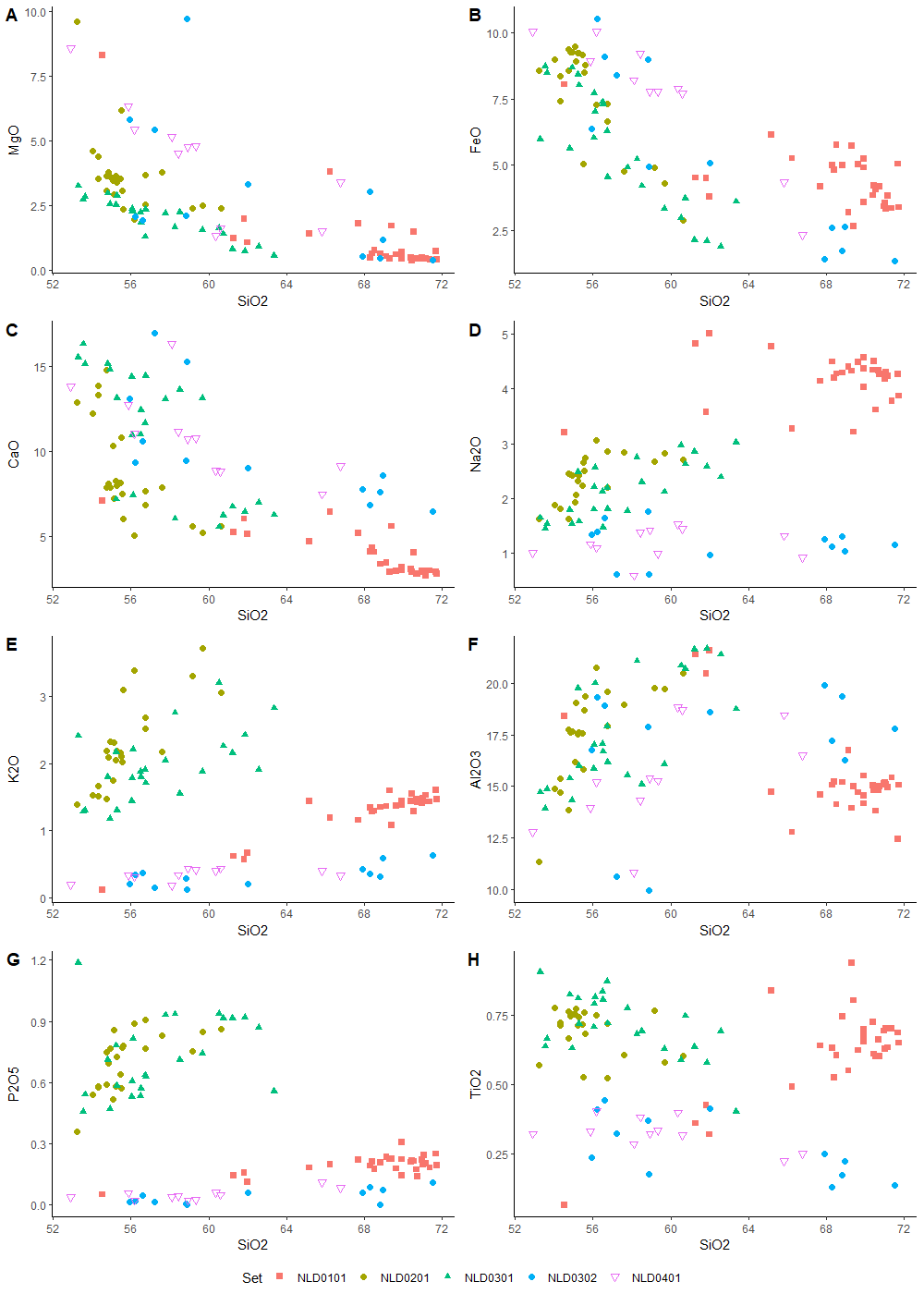


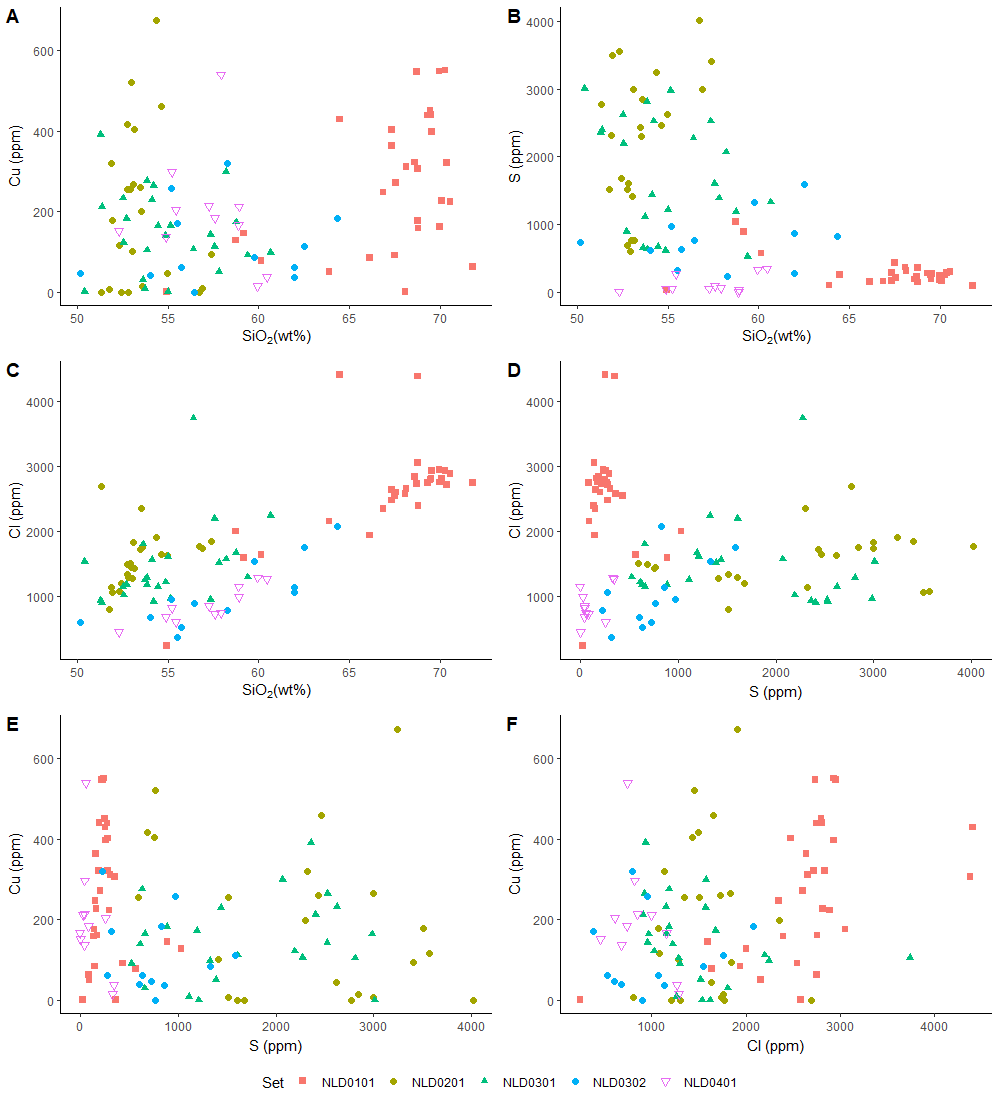


Sulfur concentrations are highest in melt inclusions below 59 wt% SiO_2_, where the concentrations drop sharply, suggesting rapid loss of sulfur either through sulfide saturation or degassing. Conversely, chlorine concentrations increase steadily with increasing SiO_2_, suggesting that chlorine degassing does not occur when sulfur concentrations drop. Copper shows a similar relationship like sulfur against SiO­_2_, with the highest concentrations occurring before 59 wt% SiO_2_ and dropping thereafter. However, instead of continuing to decrease like sulfur, the CuO concentrations exhibit a wide range in sample NLD0101, going from ~50ppm all the way up to 600 ppm. This range could arise due to the variety of ways in which Cu can be easily lost from a melt – be this from partitioning into exsolved fluid phases (which we interpret to be the case for the aforementioned sulfur decline in samples NLD0201, 0301, 0302 and 0401), or partitioning into sulfide present in the melt. Even so, the main focus should be on the ability of Cu to reach the high values of 600 ppm, showing the ability of the melt to become enriched in copper. Additionally, the high copper numbers for NLD0101 are not the result of erroneous measurement of sulfides, as they were obtained via spot analysis on the EMPA, where potential sulfide phases could be avoided under BSE view.

**REFERENCES**

1. Arculus RJ. SS200807 Voyage Summary. In: Infrastructure CNCaM, editor. CSIRO National Collections and Marine Infrastructure; 2008. pp. 1-23.

2. Danyushevsky LV, Plechov P. Petrolog3: Integrated software for modeling crystallization processes. *Geochemistry, Geophysics, Geosystems* 2011, **12**(7).

3. Le Losq C, Neuville DR, Moretti R, Roux J. Determination of water content in silicate glasses using Raman spectrometry: Implications for the study of explosive volcanism. *Am Mineral* 2012, **97**(5-6)**:** 779-790.

4. Berry AJ, Stewart GA, O'Neill HSC, Mallmann G, Mosselmans JFW. A re-assessment of the oxidation state of iron in MORB glasses. *Earth Planet Sc Lett* 2018, **483:** 114-123.

5. Berry AJ, Danyushevsky LV, St C. O’Neill H, Newville M, Sutton SR. Oxidation state of iron in komatiitic melt inclusions indicates hot Archaean mantle. *Nature* 2008, **455**(7215)**:** 960-963.

6. Burnham AD, Berry AJ, Halse HR, Schofield PF, Cibin G, Mosselmans JFW. The oxidation state of europium in silicate melts as a function of oxygen fugacity, composition and temperature. *Chem Geol* 2015, **411:** 248-259.
